# Supplementary material for: Dissecting the contributions to non-photochemical quenching in a land plant under fluctuating light
Source: Nat Commun. 2026 Mar 9;17:3664. doi: 10.1038/s41467-026-70414-2 (PMC13100187; doi:10.1038/s41467-026-70414-2)
Supplement: Supplementary file 1 — Supplementary Information [file 41467_2026_70414_MOESM1_ESM.pdf]

Supplementary Information for

Dissecting the Contributions to Non-photochemical Quenching in a Land Plant Under Fluctuating Light

Lam Lam[1,2]†, Rebecca Lee[3]†, Dhruv Patel-Tupper[2,4], Henry E. Lam[5], Tsung-Yen Lee[2,5], Alexa Ma[5], Sophia A. Ma[4], Hetty He[6], Krishna K. Niyogi[2,4,7,8]and Graham R. Fleming[1,2,5,9*]

[1]Graduate Group in Biophysics, University of California, Berkeley, CA 94720, USA.

[2]Molecular Biophysics and Integrated Bioimaging Division, Lawrence Berkeley National Laboratory, Berkeley, CA 94720, USA.

[3]Department of Chemistry, University of California, Los Angeles, CA 90095, USA.

[4]Department of Plant and Microbial Biology, University of California, Berkeley, CA 94720, USA.

[5]Department of Chemistry, University of California, Berkeley, CA 94720, USA.

[6]Department of Molecular and Cell Biology, University of California, Berkeley, CA 94720, USA.

[7]Howard Hughes Medical Institute, University of California, Berkeley, CA 94720, USA.

[8]Innovative Genomics Institute, University of California, Berkeley, CA 94720, USA.

[9]Kavli Energy Nanoscience Institute, Berkeley CA 94720, USA.

†These authors contributed equally.

*Corresponding authors. E-mail: grfleming@lbl.gov

Supplementary Figures and Tables

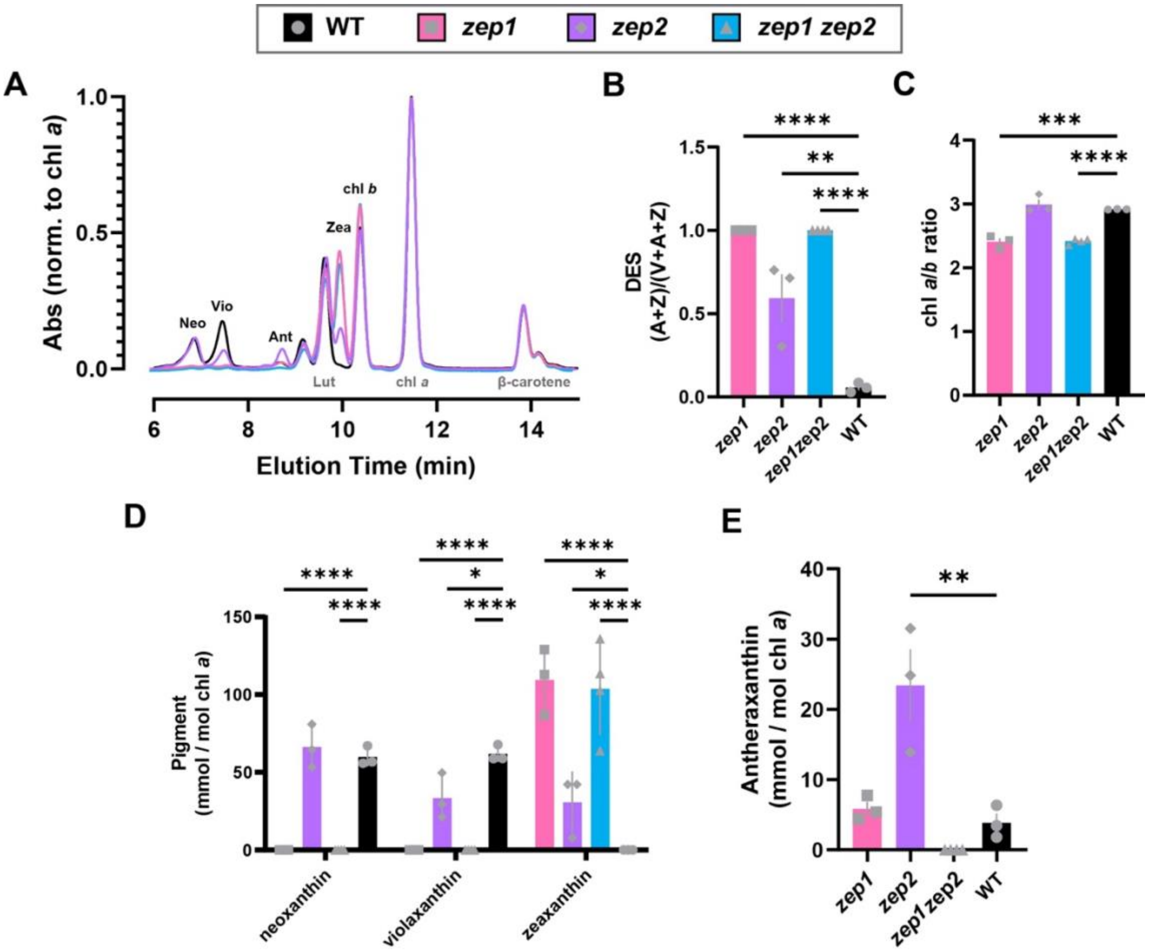

Supplementary Figure 1 Xanthophyll pigment profile of dark-acclimated *zep1*, *zep2*, and *zep1zep2* mutants.

A Overlay of representative chromatograms normalized to chlorophyll *a*, resolving neoxanthin (Neo), violaxanthin (Vio), antheraxanthin (Ant), lutein (Lut), zeaxanthin (Zea), chlorophyll *b* (Chl *b*), chlorophyll *a* (Chl *a*), and β-carotene (β-Car) after 1 hour in darkness. **B** De-epoxidation state (DES). **C** Chl *a/b* ratio. **D, E** Individual xanthophyll pigment concentrations normalized to Chl *a*, with Ant plotted on a separate y-axis to increase contrast between genotypes. Data for dark-acclimated WT (*n* = 3, black, circle) is provided as a baseline against *zep1* (*n* = 3, pink, square), *zep2* (*n* = 3, purple, diamond), and *zep1zep2* (*n* = 4, blue, triangle). Data shown as mean ± 1 SEM. Pairwise significance was determined by ordinary one-way ANOVA (B, C, E; α = 0.05) or two-way ANOVA (D, α = 0.05) using Dunnett's test for multiple comparisons against WT, with significance denoted by asterisks (**p ≤ 0.01, ***p ≤ 0.001, **p < 0.0001).

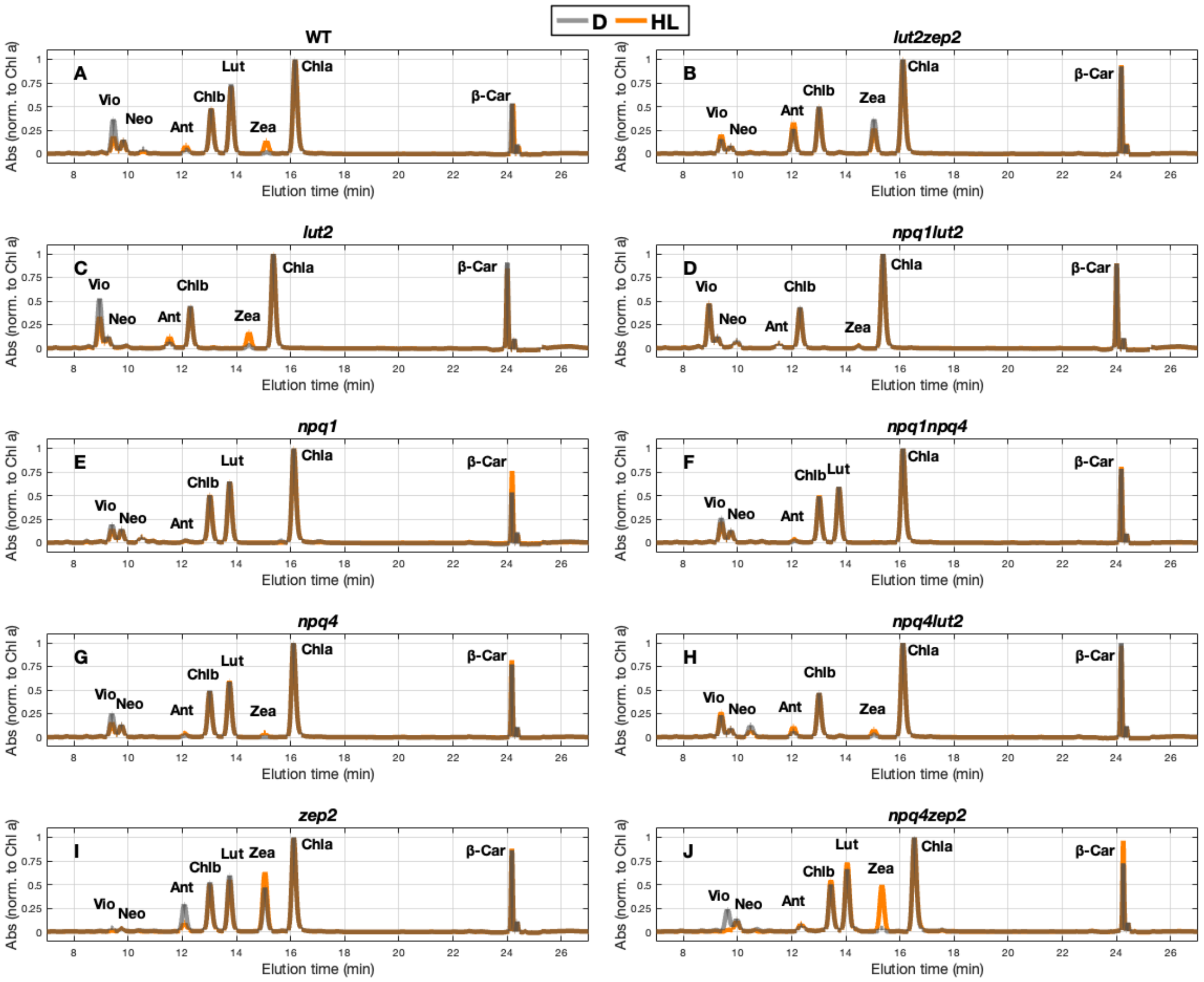

Supplementary Figure 2 Pigment profiles of *N. benthamiana* wild type (WT) and mutants.

HPLC chromatograms showing pigment composition in whole-leaf extracts from: **A** WT, **B** *zep2 lut2*, **C** *lut2*, **D** *npq1lut2*, **E** *npq1*, **F** *npq1npq4*, **G** *npq4*, **H** *npq4lut2*, **I** *zep2*, **J** *npq4zep2*. Leaves were sampled after overnight dark acclimation (D, gray line) and following 15-min exposure to high light (HL, 1500 µmol photons m^{-2} s^{-1}, orange line). Eight leaf disks (two per leaf) were pooled from four different leaves collected from three to four different plants. All chromatograms are normalized to Chl *a*. Major photosynthetic pigments are labeled at their respective elution times: Vio, Neo, Ant, Chl b, Lut, Zea, Chl *a*, β-Car.

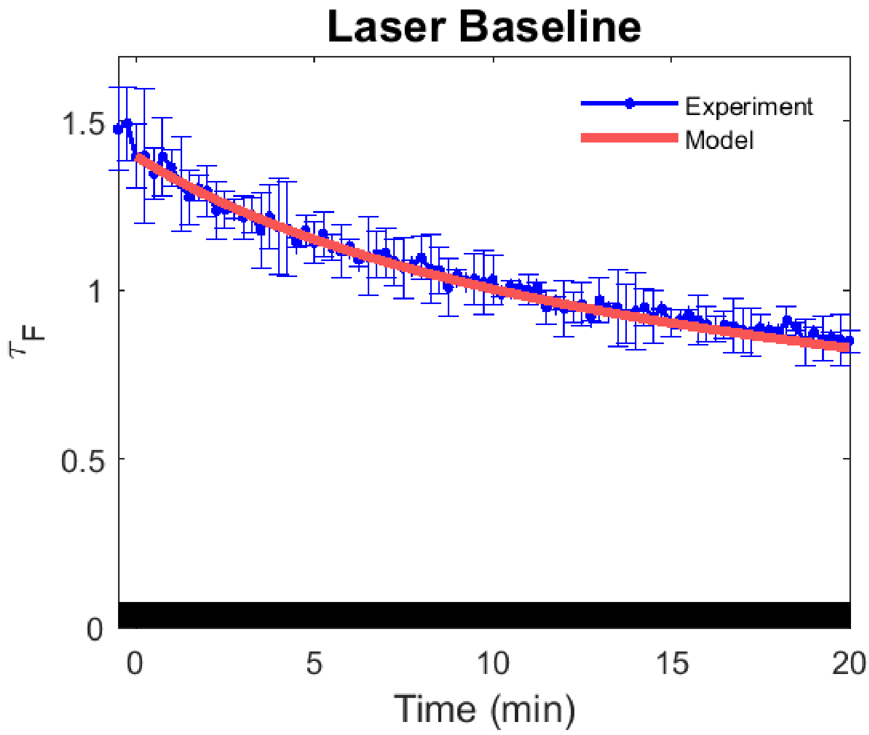

Supplementary Figure 3 Laser baseline chlorophyll fluorescence lifetime measurement on *npq1npq4* leaves (blue), and model fitting curve (red).

The model was fit to the laser baseline (no actinic light) alongside the *npq4npq1* lifetime measurements (n=3) in 20 min darkness (black bar). RMSD (s^{-1}) = 0.015. Error bars represent ± 2 SE from *n* biological replicates.

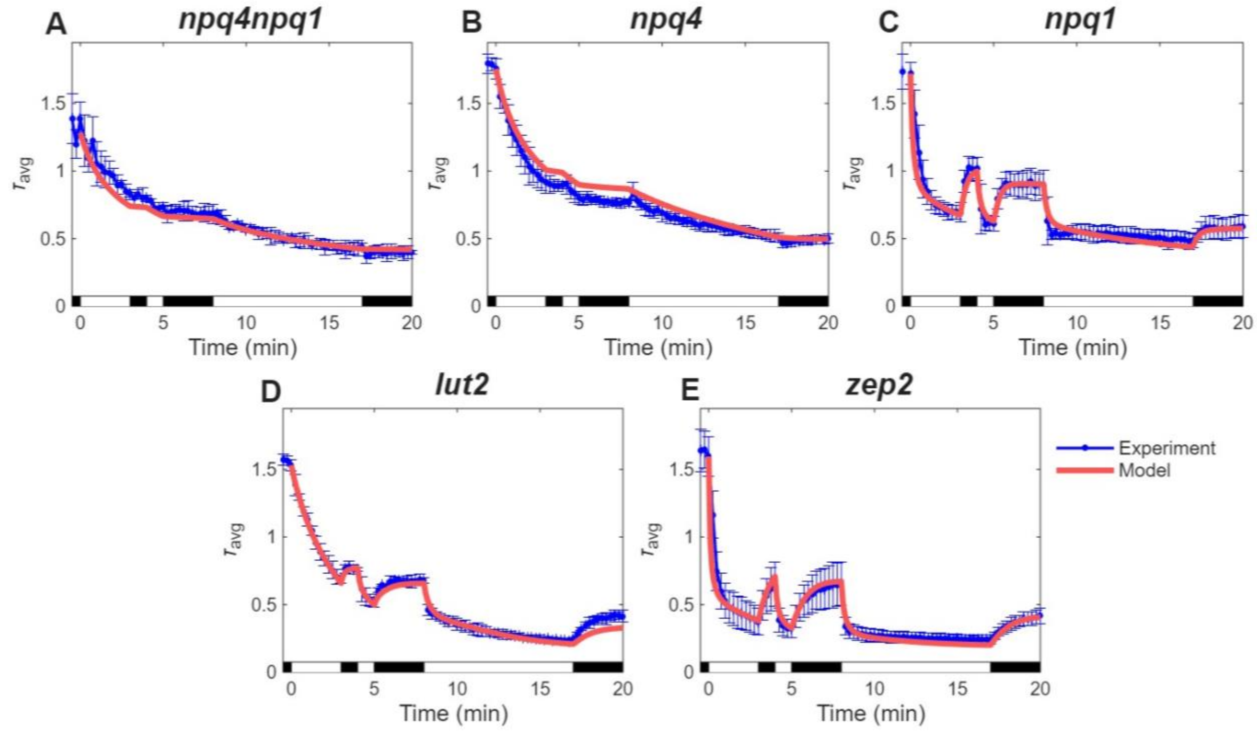

Supplementary Figure 4 Chlorophyll fluorescence lifetime measurement data (blue) and model fitting results (red) for single mutants under the 3HL–1D–1HL–3D–9HL–3D sequence.

A *npq4npq1* (n=3)*,* **B** *npq4 (n=3),* **C** *npq1* (n=6), **D** *lut2* (n=3), **E** *zep2* (n=9)**.** The 3HL–1D–1HL–3D–9HL–3D sequence was weighted equally with the 5HL-10D-5HL sequence in determination of best fit parameters. RMSD = **A** 0.049, **B** 0.105, **C** 0.115, **D** 0.264, **E** 0.404. White and black bars indicate high light (HL) and dark (D) phases of the actinic light sequence. Error bars represent ± 2 SE from *n* biological replicates.

Supplementary Table 1 Fitted parameters for NPQ kinetics for τ_F model under HL conditions.

Parameter	Value (HL)	Lower bound	Upper bound	Parameter	Value (HL)	Lower bound	Upper bound
k_{va}^{L}	2.47	2.12	3.07	k_{VDE}^{D}	0.24	0.16	0.31
k_{va}^{D}	0.014	0.012	0.015	k_{VDE}^{L}	0.28	0.26	0.31
k_{az}^{L}	0.50	0.42	0.59	$k_{av,aba1}$	0.006	0.005	0.008
k_{av}	1.12	0.99	1.23	$k_{za,aba1}$	0.038	0.024	0.057
k_{za}	0.070	0.039	0.108	$* k_{PV,f}$	1.43	1.13	1.75
$k_{PV,f}$	2.18	1.01	3.82	$* k_{PV,b}$	13.1	12.5	13.7
$k_{PV,b}$	9.43	8.29	10.51	$* k_{PA,f}$	34.4	29.5	37.3
$k_{PA,f}$	130	127	134	$* k_{PA,b}$	294	292	295
$k_{PA,b}$	254	252	257	$* k_{PZ,f}$	74.1	71.2	76.1
$k_{PZ,f}$	295	292	297	$* k_{PZ,b}$	168	165	169
$k_{PZ,b}$	126	120	132	$[V]_{0,npq1}$	49.8	49.7	49.9
$k_{QV,f}^{L}$	0.027	0.020	0.036	$[V]_{0,lut2}$	71.2	65.7	75.2
$k_{QV,b}$	0.066	0.045	0.087	$[V]_{0,npq4}$	40.6	37.5	43.9
$k_{QA,f}^{L}$	0.66	0.35	1.23	$[V]_{0,aba1}$	10.7	10.3	11.0
$k_{QA,b}$	8.57	7.45	9.32	$[V]_{0,WT}$	35.9	37.5	43.9
$k_{QZ,f}^{L}$	0.56	0.48	0.61	$[P]_{tot}$	45.4	39.9	49.1
$k_{QZ,b}$	1.22	1.09	1.34	$* [P]_{tot}$	49.9	49.7	50.0
$k_{QL,f}^{L}$	0.056	0.053	0.059				
$k_{QL,b}$	3.68	3.30	4.08				
k_{damage}^{L}	0.0222	0.0216	0.0231				
k_{damage}^{D}	0.0161	0.0155	0.0168				

Rate parameters are in units of $mmol \cdot mol\ Chl\ a^{-1} \cdot min^{-1}$. Subscripts "$f$" and "$b$" denote forward and backward rates and superscripts "L" and "D" denote light and dark conditions, respectively. Additionally, subscripts "va" and "az" denote conversion from V to A and from A to Z, while subscripts "za" and "av" denote conversion from Z to A and A to V, respectively.

Lower and upper bounds were obtained via refitting parameters to the experimental dataset 10 times and bootstrapping to estimate 95% confidence intervals. $k_{QX,f}$ is set to zero in dark conditions for all quenching species. * Denotes alternate *lut2* rates accounting for LHCII pigment composition changes. Lut concentrations were obtained from HPLC. The detailed description of the physical meanings of these parameters is provided in the Extended methods.

Supplementary Table 2 Quenching rate constants for Vio, Ant, Zea, and Lut in qE, as well as the rate constant for qZ and photoinhibition (qI).

Parameter	Value	Lower bound	Upper bound
κ_{QV}	0.040	0.032	0.046
κ_{QA}	0.174	0.131	0.202
κ_{QZ}	0.177	0.154	0.204
κ_{QL}	0.262	0.245	0.281
κ_{qZ}	0.030	0.024	0.036
κ_{qI}	3.86	3.73	3.97
† κ_{qI}	7.05	6.80	7.24

Quenching rates are a function of species concentration, expressed as $k_X = \kappa_X[X]$, where $[X]$ represents xanthophyll concentration (mmol/mol Chl a). † Denotes alternate qI quenching rate for double mutants.

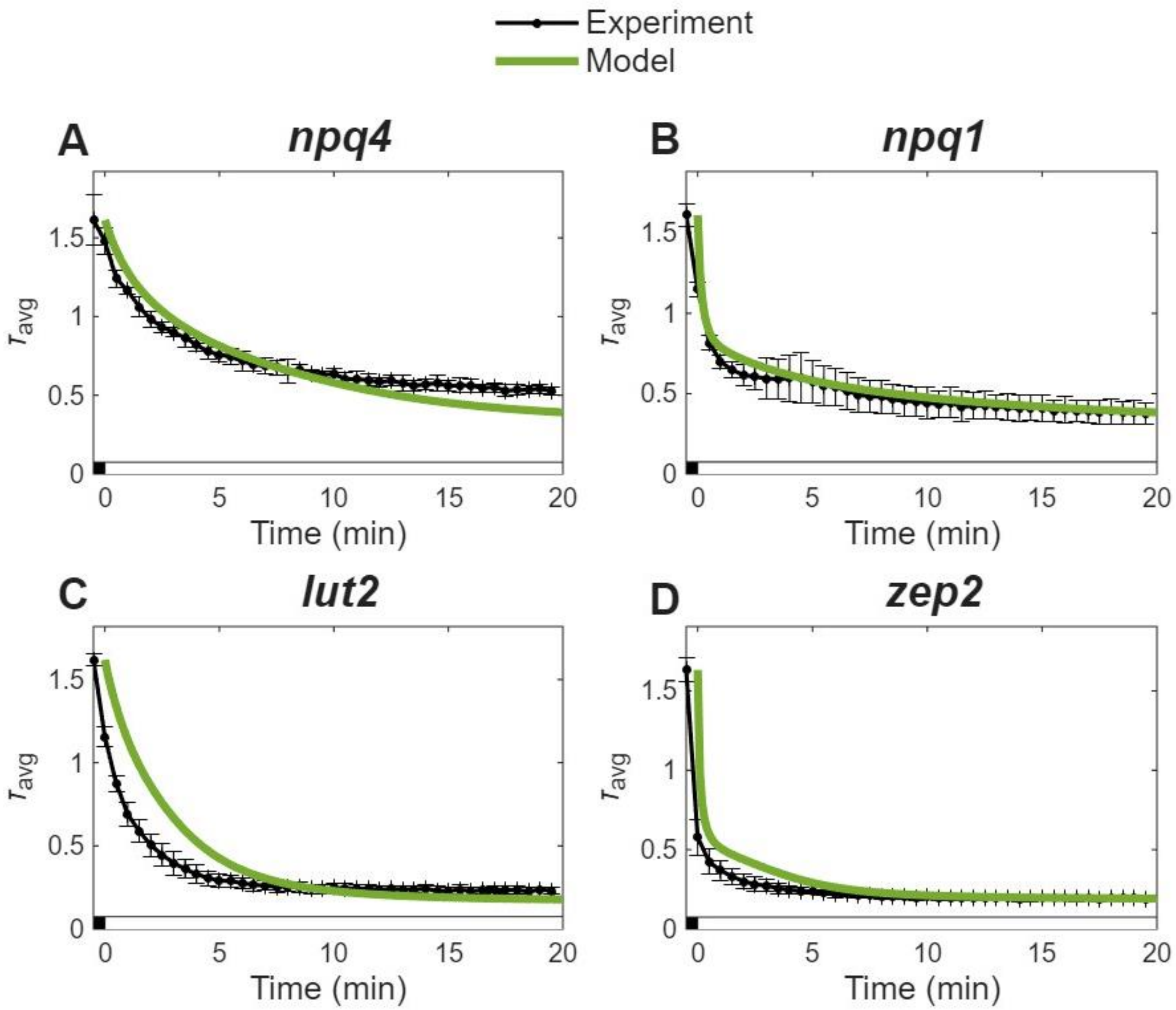

Supplementary Figure 5 Chlorophyll fluorescence lifetime measurement data (black) and model predictions (green) for single mutant sequences under the 20HL sequence.

A *npq4* (n=3), **B** *npq1* (n=4), **C** *lut2* (n=3), **D** *zep2* (n=4). Model predictions were calculated from parameters fitted in Figure 2 and Figure S4. RMSD values for the fits are (s^{-1}): **A** 0.330, **B** 0.094, **C** 0.754, **D** 0.433. A white and a black bar indicate high light (HL) and dark (D) phases of the actinic light sequence. Error bars represent ± 2 SE from *n* biological replicates.

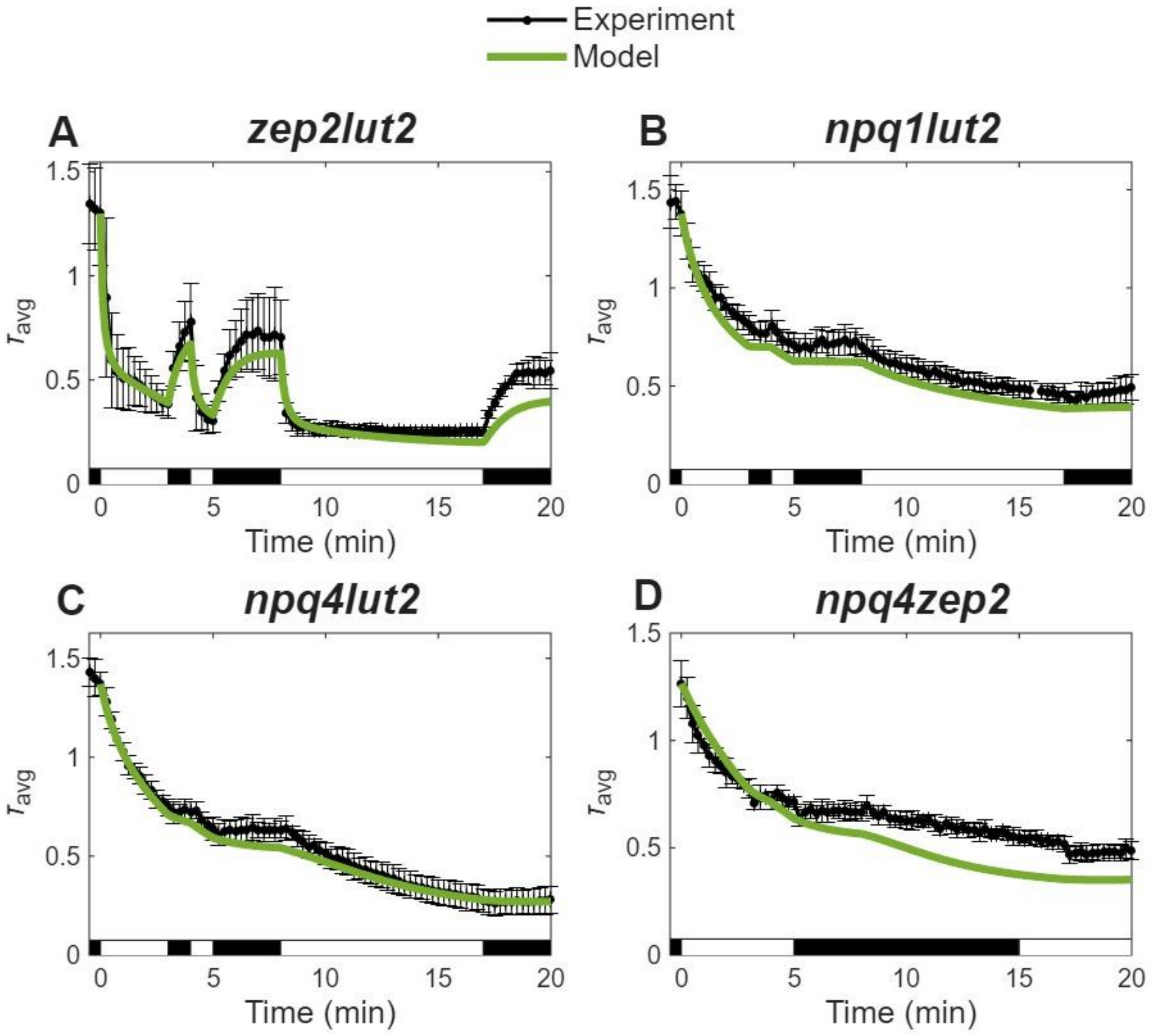

Supplementary Figure 6 Model predictions (green) and experimental validation (black) via chlorophyll fluorescence lifetime in double mutants under the 3HL-1D-1HL-3D-9HL-3D sequence.

A *zep2lut2* (*n*=3), **B** *npq1lut2* (*n*=4), **C** *npq4lut2* (*n*=4), **D** *npq4zep2* (*n*=4). Model predictions were calculated from parameters fitted in Figure 2 and Figure S4, with mutation-specific parameters combined to produce the modeled result. RMSD values for the fits are (s^{-1}) **A** 0.542, **B** 0.077, **C** 0.139 **D** 0.550. White and black bars indicate high light (HL) and dark (D) phases of the actinic light sequence. Error bars represent ± 2 SE from *n* biological replicates.

Supplementary Table 3 ***A. thaliana*** **genes, the respective** ***N. benthamiana*** **orthologs, and gRNA target sites for CRISPR-Cas9 mutagenesis.**

A. thaliana mutant	At Gene Locus	*N. benthamiana* Gene Name	Nb Genome Location	gRNA target site (from ATG)	Location	Spacer Sequences (5' -> 3')
npq4 / psbs	At1g44575	PsbS1	Niben101Scf11852g00012.1	+331 : +351	Exon 2	ACAGGTTGTACCAAAGCCAA
				+422 : +442	Exon 2	
		PsbS1	Niben101Scf05304g05008.1	+351 : +371	Exon 2	GTTGGCCGTGTTGCTATGAT
				+422 : +442	Exon 2	
npq1 / vde	At1g08550	VDE1	Niben101Scf07893g00003.1	+1983 : +2003	Exon 4	GGGAAATGGTTCATAACTCG
				+2088 : +2108	Exon 4	
		VDE1	Niben101Scf00177g07008.1	+2503 : +2523	Exon 5	TGGAGAATACGGACACCTGA
				+2608 : +2628	Exon 5	
lut2	At5g57030	LUT2-1	Niben101Scf18343g00013.1	+2105 : +2170	Exon 3	TAGTCGCCATTTACTGCACG
				+2298 : +2318	Exon 4	
		LUT2-2	Niben101Ctg13249g00004.1+ Niben101Ctg15093g00004.1*	+240 : +260	Exon 2	ATCTTAACTCGAAAGTGGAT
				+389 : +409	Exon 3	
npq2 / zep	At5g67030	ZEP1	Niben101Scf16082g00007.1	+296 : +316	Exon 1	GAAAAGGGGATTTGAGGTGT
				+474 : +494	Exon 1	
		ZEP2	Niben101Scf01553g01001.1	+296 : +316	Exon 1	GGTTTGGTTGATGGTGTTTC
				+474 : +494	Exon 1	

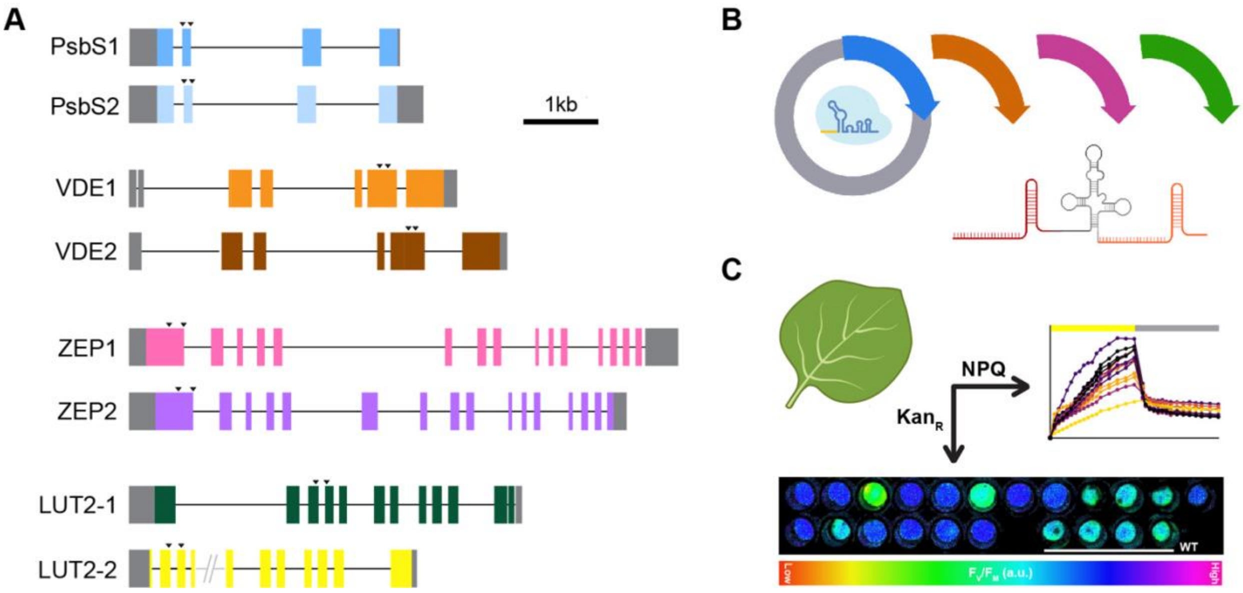

Supplementary Figure 7 Gene models and target sites for orthologous *N. benthamiana* NPQ gene mutagenesis.

A *N. benthamiana* gene models assembled from the SolGenomics draft genome of Nb-1, numbered within each pair by percent homology to their respective *A. thaliana* ortholog. Exons are shown by colored boxes, introns by black lines, and untranslated regions in gray. gRNA spacers are marked by black triangles. The gray slashes included in LUT2-2 denote separation of the gene across two draft contigs (see Supplementary Table 3). **B** Schematic for dual gRNA expression of each set of target genes using an intervening tRNA linker. **C** Screen for knockout NPQ phenotypes and sensitivity to the Cas9 selectable marker kanamycin, as shown by a decline in Fv/Fm similar to non-transgenic WT (indicated by the white line). See Methods for more details.

Supplementary Table 4 Primers for genomic DNA amplification of *N. benthamiana ZEP* genes.

Oligo ID	Oligo (5' -> 3')	Fragment Size	Tm
**oNb11 ZEP_1.F**	CCTCATTGGTTCAGCTTC	806 bp	58
**oNb12 ZEP_1.R**	GACATGCAGTATTAAATGACACC		
**oNb13 ZEP_2.F**	GGATTTCCCGACAGAGTTG	530 bp	58
**oNb14 ZEP_2.R**	GCTTGTGTATGATTGTTACAGTGC		

Supplementary Table 5 Homozygous, Cas9-free *ZEP* knockout alleles of *N. benthamiana*

T_0 Parent	Homozygous Mutant ID	*ZEP1*	*ZEP2*	Genotype	Mutant Label
ZEP_ko-4	38, 44, 58	+1 bp	-6 bp	*zep1*	*zep1*
ZEP_ko-4	18, 39, 63	-3 bp	-8 bp	*zep2*	*zep2*
ZEP_ko-4	46*, 68*, 72*, 88*	+1 bp	-8 bp	*zep1zep2*	*zep1zep2*

Column 2 lists the homozygous, Cas9-free mutant line ID. Columns 3–4 report the mutation at each targeted locus (*ZEP1* and *ZEP2*); values are indel sizes (bp) relative to WT at the designed gRNA target site (+, insertion; -, deletion; WT, no mutation). Column 6 provides the mutant label used in this study. * Indicates progeny that did not survive to seed set.

Supplementary Table 6 Homozygous, Cas9-free higher-order mutants of *N. benthamiana* isolated by genetic crosses

Genetic Cross	Homozygous Mutant ID	***PSBS1***	***PSBS2***	***VDE1***	***VDE2***	Genotype	Mutant Label
npq1 x *npq4*	#27-4-24	-95 bp	+1bp	-5 bp	-60bp	*psbs1 psbs2 vde1 vde2*	*npq1npq4*
		PSBS1	***PSBS2***	***LUT2-1***	***LUT2-2***		
npq4 x *lut2*	#25	+1bp	+1bp	-3 bp	+1bp	*psbs1 psbs2 lut2-1 lut2-2*	*npq4lut2*
		PSBS1	***PSBS2***	***ZEP1***	***ZEP2***		
npq4 x *zep2*	#72-15	-8bp	-4 bp	-3bp/<br>WT	-8 bp	*psbs1 psbs2 zep2*	*npq4zep2*
		VDE1	***VDE2***	***LUT2-1***	***LUT2-2***		
npq1 x *lut2*	#3-2, #3-20	-11 bp	+1bp	+1bp	+1bp	*vde1 vde2 lut2-1 lut2-2*	*npq1lut2*
		ZEP1	***ZEP2***	***LUT2-1***	***LUT2-2***		
zep2 x *lut2*	#2-7	-3bp	-8bp	+1bp	+1bp	*zep2 lut2-1 lut2-2*	*zep2lut2*

Column 2 lists the homozygous, Cas9-free mutant line ID. Columns 3–6 report the mutation at each targeted locus (e.g., *PSBS1*/*PSBS2*/*VDE1*/*VDE2*, etc.); values are indel sizes (bp) relative to WT at the designed gRNA target site (+, insertion; -, deletion; WT, no mutation). Column 8 provides the mutant label used in this study.

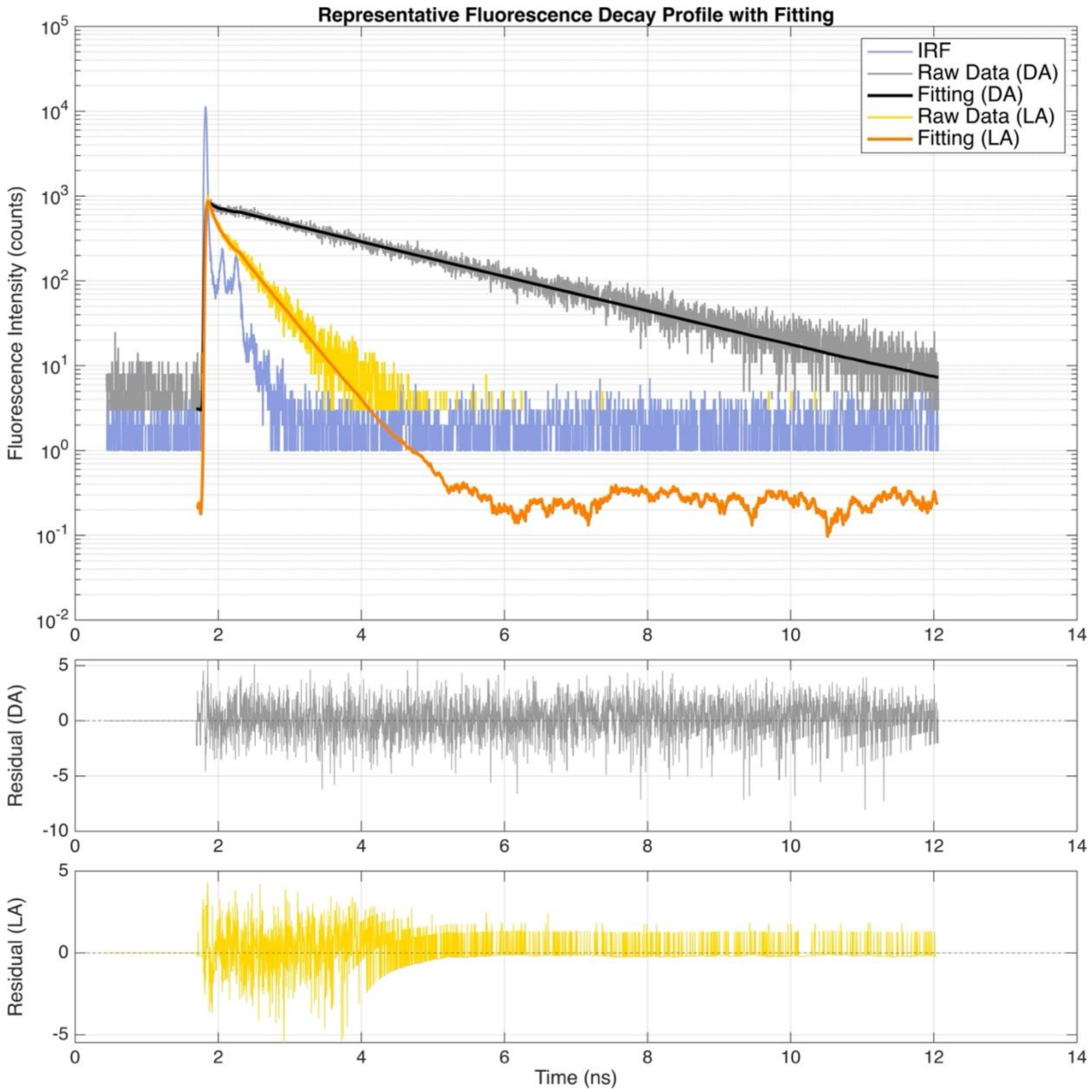

Supplementary Figure 8 Representative plots of chlorophyll fluorescence lifetime raw data, fitting, and instrument response function (IRF).

The main panel shows semi-logarithmic plots of the IRF (blue) and raw fluorescence decay traces for a representative dark-acclimated (DA, grey) and 20-minute light-acclimated (LA, yellow) WT leaf sample, along with their respective bi-exponential fits (DA, black; LA, orange). The data represent a 0.2-second timestep extracted from a single fluorescence snapshot. Residuals (differences between the raw data and the fits) for the DA and LA traces are shown in the lower subpanels, illustrating the quality of the fits.

Supplementary Methods

Our kinetic model incorporates three key components: a dynamic xanthophyll cycle, lutein activation, and a simplified phenomenological representation of qI. To clarify the conceptual framework of this model, we summarize below the key assumptions that were required (or not required) for its implementation and interpretation. This overview is intended to guide readers in understanding the model's scope and limitations.

Model Assumptions

Assumptions Needed

1. NPQ in plants is mainly contributed by qE (VAZ, and lutein), qZ, and qI. In our study, qT, qH, and chloroplast movement have negligible effects.
2. Each NPQ contribution contributes independently to the Chl fluorescence quenching, and can be therefore modelled separately.
3. Quenching is modeled using a Stern-Volmer–like relation between fluorescence lifetime and the concentrations of quencher molecules (QV, QA, QZ, QL, qI). The Stern-Volmer relation has been the standard method of modeling fluorescence quenching since its introduction in 1919[45].
4. qZ depends on the concentration of Zea in a light-independent manner.
5. Lut is assumed to be always protein-bound when present.
6. The activation of protein-xanthophyll complexes (PX) to quenching complexes (QX) is needed for the qE type of quenching to be activated. While the total pool of xanthophyll-

binding sites P is fixed and shared among Zea, Vio, Ant, and Lut, the formation/dissociation kinetic rates are unique to each xanthophyll (Zea, Vio, Ant, Lut).

[qI Model]

7. The qI component is modeled phenomenologically as irreversible photodamage to PSII reaction centers, accumulating over time with a rate proportional to excitation (i.e., inversely to lifetime).

8. The reverse/repair process is assumed to be negligible within our timescale.

9. Quenching observed during laser-only exposure is attributed to qI and is assumed to be sufficiently captured by fitting qI parameters in *npq4npq1* data.

[Data Validation]

10. Time-resolved fluorescence snapshots are assumed to sufficiently capture the dynamics of all the NPQ contributions, with average lifetimes representing total quenching at each time point. The average lifetime is directly proportional to the fluorescence quantum yield. We have shown elsewhere that fluorescence decays of a model thylakoid membrane[57] containing roughly 30,000 rates can be fit very well with two exponentials, but those two components do not relate to the underlying microscopic dynamics in any direct way.

[*lut2* specifically]

11. Considering the structural changes in the absence of lutein, the number of protein-xanthophyll binding sites ([P]_tot) is assumed to be different between WT and the *lut2* mutant. [P]_tot in *lut2* was approximated by modeling to be ~50% of [P]_tot in WT.

Assumptions Not Needed

1. By fitting the lifetime values directly, we do not assume zero initial quenching in all the mutants as was done by Short et al.[37].This is important for mutants such as *zep2*.
2. Despite utilizing some widely accepted biochemical processes[6] involved in induction of qE (including the VAZ cycle), the model does not make assumptions on the actual physical mechanism of how these quenching processes happen. The model never claims to resolve ultrafast excited-state dynamics. For example, the model doesn't assume direct quenching (by energy transfer/charge transfer) of Chl excited states by Car.
3. The model does not resolve or define the exact mechanistic basis of qZ or qI. It only differentiates them kinetically from qE based on mutant behavior and pigment profiles.
4. Calculating per-molecule quenching efficiencies does not reflect direct mechanistic interpretation. It reflects the general impact on NPQ/lifetime caused by each species.

[Other NPQ components]

5. While qT mutants were tested (e.g., *stn7* mutants) and found to have negligible influence on our system over the timescale we studied, we do not reject or adopt any specific quenching mechanism. We do not claim these (qT, qH, Chloroplast movement/thylakoid membrane structural adjustment) are irrelevant to NPQ in general.

[Fitting Interpretation]

6. The model does not assume that NPQ component contributions (e.g., qE, qZ, qI) are additive in general. However, when making predictions, the model does compute overall NPQ as a sum of individual quenching components (see Assumption 2 - each mechanism

is independent). However, our approach of fitting simpler mutants and directly applying their rates to complex genotypes, such as *zep2* and WT, with great accuracy shows that NPQ components can be considered as additive.

7. The model captures most of the NPQ dynamics in different illumination sequences. We designed different irregular sequences that presented a more difficult challenge for the model to make predictions.

Model Structure and Fitting Procedures

Based on the assumptions outlined above, we constructed a kinetic model to quantitatively capture the contributions of different NPQ components under dynamic illumination conditions. Below, we describe the framework of the model and the procedures used for parameter fitting. In this model as mentioned above, we assume xanthophylls (X= Vio, Ant, Zea, and Lut) all have some quenching capacity defined by their ability to (1) bind to an antenna complex, forming "PX", (2) activate under light conditions, forming a quencher "QX", and (3) dissipate excitation energy at some rate (κ_{QX}). We assume Lut is always protein-bound, i.e. always in a PL or QL state. The xanthophyll cycle is mediated by the enzyme VDE, which exists in an active protonated (VDE_a) and de-protonated (VDE_i) state. qI is phenomenologically modeled as the irreversible conversion of functional PSII reaction centers I to a damaged state I*, representing photoinhibition. In total, the model incorporates 13 chemical species. Explicitly, the kinetic scheme is as follows:

$$V + P \rightleftharpoons^{k_{PV,f}}{}_{k_{PV,b}} PV \rightleftharpoons^{k_{QV,f}^{L/D}}{}_{k_{QV,b}} QV \tag{S.1}$$

$$A + P \rightleftharpoons^{k_{PA,f}}{}_{k_{PA,b}} PA \rightleftharpoons^{k_{QA,f}^{L/D}}{}_{k_{QA,b}} QA \tag{S.2}$$

$$Z + P \rightleftharpoons^{k_{PZ,f}}{}_{k_{PZ,b}} PZ \rightleftharpoons^{k_{QZ,f}}{}_{k_{QZ,b}} QZ \tag{S.3}$$

$$V + VDE_a \rightarrow^{k_{va}} \ A + VDE_a \rightarrow^{k_{az}} \ Z + VDE_a \tag{S.4}$$

$$Z \rightarrow^{k_{za}} \ A \rightarrow^{k_{av}} \ V \tag{S.5}$$

$$PL \rightleftharpoons^{k_{QL,f}^{\frac{L}{D}}}{}_{k_{QL,b}} QL \tag{S.6}$$

$$I \rightarrow^{k_{damage}^{L/D}} \ I^{*} \tag{S.7}$$

Where subscripts "*f*" and "*b*" denote forward and backward rates and superscripts "*L*" and "*D*" denote light and dark conditions, respectively. Additionally, subscripts "*va*" and "*az*" denote conversion from V to A and from A to Z, while subscripts "*za*" and "*av*" denote conversion from Z to A and A to V, respectively. To obtain the model's kinetic differential

equation system, each step above is treated as an elementary reaction with rates as given. Using first-order rate kinetics, differential equations are constructed for each species as follows:

$$\frac{d}{dt}[V] = k_{av}[A] - k_{va}[VDE_a][V] - k_{PV,f}[V][P] + k_{PV,b}[PV] \tag{S.8}$$

$$\frac{d}{dt}[A] = k_{va}[VDE_a][V] + k_{za}[Z] - k_{az}[VDE_a][A] - k_{av}[A] - k_{PA,f}[A][P] + k_{PA,b}[PA] \tag{S.9}$$

$$\frac{d}{dt}[Z] = k_{az}[VDE_a][A] - k_{za}[Z] - k_{PZ,f}[Z][P] + k_{PZ,b}[PZ] \tag{S.10}$$

$$\frac{d}{dt}[PX] = k_{PX,f}[X][P] - k_{PX,b}[PX] - {k_{QX,f}}^{L/D}[PX] + k_{QX,b}[QX] \tag{S.11}$$

where $X = V, A, Z$

$$\frac{d}{dt}[PL] = k_{QL,b}[QL] - {k_{QL,f}}^{L/D}[PL] \tag{S.12}$$

$$\frac{d}{dt}[QX] = {k_{QV,f}}^{L/D}[PX] - k_{QV,b}[QX] \tag{S.13}$$

where $X = V, A, Z$

$$\frac{d}{dt}[\alpha_{qI}] = {k_{damage}}^{L/D}\tau_F(1 - \alpha_{qI}) \tag{S.14}$$

$$\frac{d}{dt}[\alpha_{VDE}] = k_{VDE}^{L/D}\left(\alpha_{vde,eq}^{L/D} - \alpha_{vde}\right) \tag{S.15}$$

Light/dark (L/D) dependent rate constants take different values depending on light conditions at time *t* during the sequence of light exposures. These values are fit independently, with $k^{L/D} \equiv k^{L/D}(t) = k^L$ if HL at time *t*, or k^D if D at time *t*. We set ${k_{QX,f}}^D$ to zero, assuming zero QX activation in dark.

To reduce the number of parameters needed to fit the model, we take VDE concentration as a ratio: $\alpha_{vde_a}(t) = \frac{[VDE_a]}{[VDE^L_{a,eq}]}$ where subscript "eq" denotes the equilibrium condition. We then approximate the value $k_{va/az}[VDE](t) = k^L_{va/az,max} \cdot \alpha_{vde_a}(t)$. We further model α_{vde} as a dynamic variable, with an equilibrium value of $\alpha^L_{vde,eq} = 1$ in light conditions, and $\alpha^D_{vde,eq} = \frac{k^D_{va,max}}{k^L_{va,max}}$ under dark conditions:

$$\frac{d\alpha_{vde}}{dt} = k^{L/D}_{VDE}\left(\alpha^{L/D}_{vde,eq} - \alpha_{vde}\right) \tag{S.16}$$

which simplifies xanthophyll cycle dynamics. We approximate $k_{va/az}(t) = k^{L/D}_{va/az,max} \cdot \alpha_{vde_a}(t)$ and directly fit the maximum rates $k^{L/D}_{va/az,max}$ in the model. Similarly, the rate of damage is modeled as a ratio of damaged cores to the whole pool: $\alpha_{qI}(t) = \frac{I^*}{I+I^*}$. Photoinhibition is thus modeled as the irreversible accumulation of α_{qI}, whose rate is proportional to τ_F:

$$\frac{d\alpha_{qI}}{dt} = k^{L/D}_{damage} * \left(1 - \alpha_{qI}\right) * \tau_F \tag{S.17}$$

Hence a decrease in lifetime (as defined below, within the model) decreases the rate of photoinhibition.

The complete model can predict dynamic concentrations of the various carotenoid quenchers, enzyme activation kinetics, and the rate and extent of damage from photoinhibition. Model parameters are quantified using a least squares fit of experimental data to a calculated Chl *a* fluorescence lifetime. The time-dependent expression for τ_F is:

$$\tau_F(t) = \frac{1}{k_{r,nr} + \sum_X \kappa_{QX}[QX] + \kappa_{qZ}[Z] + \kappa_{qI}\alpha_{qI}} \tag{S.18}$$

The derivation of this formula is presented in Results and reproduced below.

The total fluorescence decay rate, $\tau_F(t)$, is determined by several competing processes, including the non-radiative decay and the intrinsic fluorescence decay of Chl a, qE, qZ, and qI. Assuming the quenching rate is linearly dependent on the concentration of quenching species, we define the fluorescence lifetime of Chl a via a Stern-Vollmer type approach as:

$$\frac{1}{\tau_F} = \kappa_{r,nr} + \kappa_{qE}[QX] + \kappa_{qZ}[Z] + \kappa_{qI}[I] \quad \text{(S.19)}$$

Where $\kappa_{qE,}$ $\kappa_{qZ,}$ κ_{qI} represent the quenching rate constants of qE, qZ, and qI, respectively, and $\kappa_{r,nr}$ accounts for all the other radiative and non-radiative de-excitation processes. Furthermore, since QV, QA, QZ, and QL may exhibit different per-molecule quenching efficiencies, we further distinguish their contributions within qE:

$$k_{qE}[QX] \approx \kappa_{QV}[QV] + \kappa_{QA}[QA] + \kappa_{QZ}[QZ] + \kappa_{QL}[QL] = \sum_X \quad \kappa_{QX}[QX] \quad \text{(S.20)}$$

This yields the final time-dependent expression (S.18) for the Chl a fluorescence lifetime:

$$\tau_F(t) = \frac{1}{k_{r,nr} + \sum_X \kappa_{QX}[QX] + \kappa_{qZ}[Z] + \kappa_{qI}\alpha_{qI}}$$

This formulation allows for the direct quantification of quenching rates and per-molecule efficiencies of the individual quenchers while accounting for genotype-dependent variations in τ_{dark} $(t = 0)$. In this model, $\kappa_{qX,}$ $\kappa_{qZ,}$ κ_{qI} are additional fitting parameters representing the rate of Chl a exciton decay per molecule of quencher (s^{-1}(mmol/mol Chl a)$^{-1}$). $k_{r,nr}$ is calculated directly from τ_{dark} $(t = 0)$ for each mutant dataset as $\frac{1}{\tau_F(0)} - \kappa_{qZ}[Z]_0$, assuming zero initial qE quenching, zero initial qI, and qZ calculated from initial zeaxanthin concentration. Thus, the model always requires an input estimate of τ_{dark}. For genotypes with standard NPQ recovery capabilities, $[Z]_0 \approx 0$, so the expression simplifies to $\frac{1}{\tau_F(0)}$.
