# Supplementary material for: Dissecting the contributions to non-photochemical quenching in a land plant under fluctuating light
Source: Nat Commun. 2026 Mar 9;17:3664. doi: 10.1038/s41467-026-70414-2 (PMC13100187; doi:10.1038/s41467-026-70414-2)
Supplement: Supplementary file 2 — Description of Additional Supplementary Information [file 41467_2026_70414_MOESM2_ESM.docx]

**Supplementary Movie 1 Time-resolved NPQ component contributions during 20 min continuous high light (20HL).**

Stacked bars show the contributions of qZ, qI, and qE subcomponents (qE_L_, qE_V_, qE_A,_ qE_Z_) in *npq4npq1*, *npq4*, *npq1*, *lut2*, *zep2*, and WT over time under the 20HL actinic light sequence. NPQ contributions were quantified component-wise by the linear relation $\kappa_{qX}\cdot QX(t)$ for each quenching xanthophyll *QX*, $\kappa_{qZ}\cdot Z(t)$ for qZ, and $\kappa_{qI}\cdot\alpha_{qI}(t)$ for qI. Yellow background indicates HL illumination; grey background indicates darkness. The sum of NPQ contributions may not be linearly proportional to overall Chl* quenching.

**Supplementary Movie 2 Time-resolved NPQ component contributions under the 5HL-10D-5HL actinic light sequence.**

Stacked bars show the contributions of qZ, qI, and qE subcomponents (qE_L_, qE_V_, qE_A,_ qE_Z_) in *npq4npq1*, *npq4*, *npq1*, *lut2*, *zep2*, and WT over time under the 5HL-10D-5HL sequence. NPQ contributions were quantified component-wise by the linear relation $\kappa_{qX}\cdot QX(t)$ for each quenching xanthophyll *QX*, $\kappa_{qZ}\cdot Z(t)$ for qZ, and $\kappa_{qI}\cdot\alpha_{qI}(t)$ for qI. Yellow background indicates HL illumination; grey background indicates darkness. The sum of NPQ contributions may not be linearly proportional to overall Chl* quenching.
