# Supplementary figures and images for: Dissecting the contributions to non-photochemical quenching in a land plant under fluctuating light
Source: Nat Commun. 2026 Mar 9;17:3664. doi: 10.1038/s41467-026-70414-2 (PMC13100187; doi:10.1038/s41467-026-70414-2)

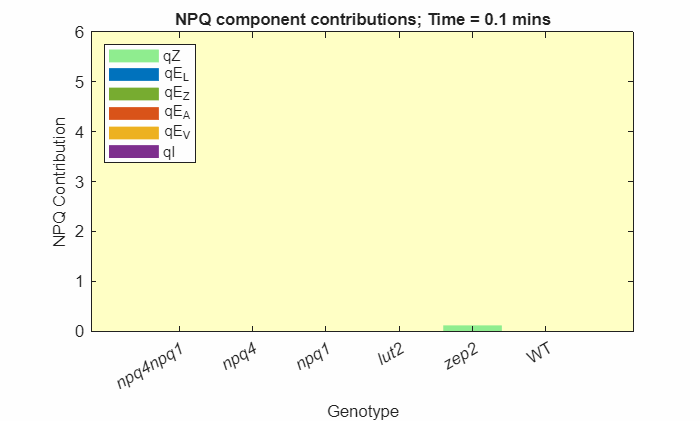

Supplement: Supplementary file 3 — Supplementary Movie 1 [file 41467_2026_70414_MOESM3_ESM.gif]

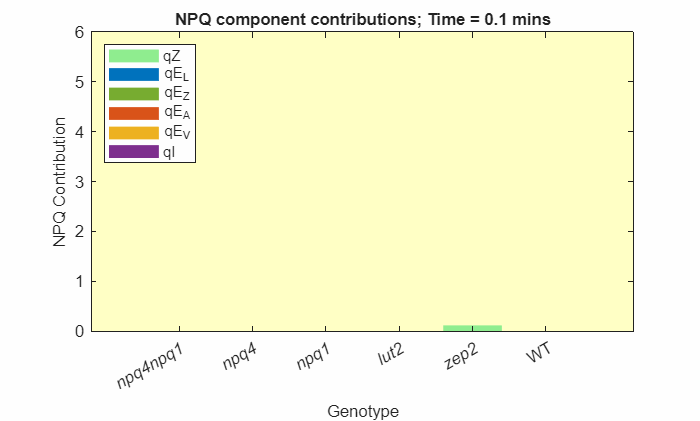

Supplement: Supplementary file 4 — Supplementary Movie 2 [file 41467_2026_70414_MOESM4_ESM.gif]
