# Supplementary material for: Dissecting the contributions to non-photochemical quenching in a land plant under fluctuating light
Source: Nat Commun. 2026 Mar 9;17:3664. doi: 10.1038/s41467-026-70414-2 (PMC13100187; doi:10.1038/s41467-026-70414-2)
Supplement: Supplementary file 5 — Reporting Summary [file 41467_2026_70414_MOESM5_ESM.pdf]

nature portfolio

Corresponding author(s): Graham Fleming

Last updated by author(s): Feb 17, 2026

Reporting Summary

Nature Portfolio wishes to improve the reproducibility of the work that we publish. This form provides structure for consistency and transparency in reporting. For further information on Nature Portfolio policies, see our Editorial Policies and the Editorial Policy Checklist.

Statistics

For all statistical analyses, confirm that the following items are present in the figure legend, table legend, main text, or Methods section.

n/a	Confirmed	
☐	☒	The exact sample size (*n*) for each experimental group/condition, given as a discrete number and unit of measurement
☐	☒	A statement on whether measurements were taken from distinct samples or whether the same sample was measured repeatedly
☒	☐	The statistical test(s) used AND whether they are one- or two-sided<br>*Only common tests should be described solely by name; describe more complex techniques in the Methods section.*
☒	☐	A description of all covariates tested
☒	☐	A description of any assumptions or corrections, such as tests of normality and adjustment for multiple comparisons
☒	☐	A full description of the statistical parameters including central tendency (e.g. means) or other basic estimates (e.g. regression coefficient) AND variation (e.g. standard deviation) or associated estimates of uncertainty (e.g. confidence intervals)
☒	☐	For null hypothesis testing, the test statistic (e.g. *F*, *t*, *r*) with confidence intervals, effect sizes, degrees of freedom and *P* value noted<br>*Give P values as exact values whenever suitable.*
☒	☐	For Bayesian analysis, information on the choice of priors and Markov chain Monte Carlo settings
☒	☐	For hierarchical and complex designs, identification of the appropriate level for tests and full reporting of outcomes
☒	☐	Estimates of effect sizes (e.g. Cohen's *d*, Pearson's *r*), indicating how they were calculated

Our web collection on statistics for biologists contains articles on many of the points above.

Software and code

Policy information about availability of computer code

Data collection	LabView 86, Matlab 2023b
Data analysis	Matlab 2023b, Excel

For manuscripts utilizing custom algorithms or software that are central to the research but not yet described in published literature, software must be made available to editors and reviewers. We strongly encourage code deposition in a community repository (e.g. GitHub). See the Nature Portfolio guidelines for submitting code & software for further information.

Data

Policy information about availability of data

All manuscripts must include a data availability statement. This statement should provide the following information, where applicable:

- Accession codes, unique identifiers, or web links for publicly available datasets
- A description of any restrictions on data availability
- For clinical datasets or third party data, please ensure that the statement adheres to our policy

Source data are provided with this paper. The data supporting the findings of this study are available at https://doi.org/10.5281/zenodo.16755870.

Research involving human participants, their data, or biological material

Policy information about studies with human participants or human data. See also policy information about sex, gender (identity/presentation), and sexual orientation and race, ethnicity and racism.

Reporting on sex and gender	n/a
Reporting on race, ethnicity, or other socially relevant groupings	n/a
Population characteristics	n/a
Recruitment	n/a
Ethics oversight	n/a

Note that full information on the approval of the study protocol must also be provided in the manuscript.

Field-specific reporting

Please select the one below that is the best fit for your research. If you are not sure, read the appropriate sections before making your selection.

[x] Life sciences [ ] Behavioural & social sciences [ ] Ecological, evolutionary & environmental sciences

For a reference copy of the document with all sections, see nature.com/documents/nr-reporting-summary-flat.pdf

Life sciences study design

All studies must disclose on these points even when the disclosure is negative.

Sample size	Leaves were selected from various plants and batches, numbers of measurements were selected to ensure a reproducible set of data could be obtained with reasonable error bar.
Data exclusions	n/a
Replication	Biological replicates were used in this study, data were reproducible within reasonable error bars.
Randomization	Biological replicates from various plants and batches were selected.
Blinding	n/a

Reporting for specific materials, systems and methods

We require information from authors about some types of materials, experimental systems and methods used in many studies. Here, indicate whether each material, system or method listed is relevant to your study. If you are not sure if a list item applies to your research, read the appropriate section before selecting a response.

Materials & experimental systems

n/a	Involved in the study	
[x]	[ ]	Antibodies
[x]	[ ]	Eukaryotic cell lines
[x]	[ ]	Palaeontology and archaeology
[x]	[ ]	Animals and other organisms
[x]	[ ]	Clinical data
[x]	[ ]	Dual use research of concern
[ ]	[x]	Plants

Methods

n/a	Involved in the study	
[x]	[ ]	ChIP-seq
[x]	[ ]	Flow cytometry
[x]	[ ]	MRI-based neuroimaging

Plants

Seed stocks	Transgenic Nicotiana benthamiana (accession Nb-1) lines were generated via Agrobacterium-mediated transformation by the Ralph M. Parsons Foundation Plant Transformation Facility at UC Davis (https://ptf.ucdavis.edu/).
Novel plant genotypes	Genomic DNA from putative knockout lines was isolated and genotyped by Phire Plant Direct PCR Master Mix (ThermoScientific™, Catalog #F160L) using the supplied dilution buffer. DNA was amplified by PCR using primers that spanned the two gRNA target sites for each gene of interest, with primer pairs specific to one of the two highly similar paralogs. PCR products were purified and sequenced by Sanger sequencing using the primers listed in Table S4. The knockout alleles for single trait and higher order mutants are listed in Table S5 and Table S6, respectively. Segregation of the Cas9 transgene was determined via changes in chlorophyll fluorescence after antibiotic treatment as previously described.
Authentication	PCR tests, PAM fluorometry, HPLC pigment concentration tests as well as Chl fluorescence lifetime measurements were conducted to confirm the new genotype lines.
